# Using a water-immiscible ionic liquid to improve asymmetric reduction of 4-(trimethylsilyl)-3-butyn-2-one catalyzed by immobilized *Candida parapsilosis *CCTCC M203011 cells

**DOI:** 10.1186/1472-6750-9-90

**Published:** 2009-10-22

**Authors:** Wen-Yong Lou, Lei Chen, Bo-Bo Zhang, Thomas J Smith, Min-Hua Zong

**Affiliations:** 1 State Key Laboratory of Pulp and Paper Engineering, College of Light Industry & Food Sciences, South China University of Technology, Guangzhou 510640, PR China; 2 Biomedical Research Centre, Sheffield Hallam University, Owen Building, Howard Street, Sheffield, S1 1WB, UK

## Abstract

**Background:**

Whole cells are usually employed for biocatalytic reduction reactions to ensure efficient coenzyme regeneration and to avoid problems with enzyme purification and stability. The efficiency of whole cell-catalyzed bioreduction is frequently restricted by pronounced toxicity of substrate and/or product to the microbial cells and in many instances the use of two-phase reaction systems can solve such problems. Therefore, we developed new, biphasic reaction systems with biocompatible water-immiscible ionic liquids (ILs) as alternatives to conventional organic solvents, in order to improve the asymmetric reduction of 4-(trimethylsilyl)-3-butyn-2-one (TMSB) to (*S*)-4-(trimethylsilyl)-3-butyn-2-ol {(*S*)-TMSBOL}, a key intermediate for synthesis of 5-lipoxygenase inhibitors, using immobilized *Candida parapsilosis *CCTCC M203011 cells as the biocatalyst.

**Results:**

Various ILs exerted significant but different effects on the bioreduction. Of all the tested water-immiscible ILs, the best results were observed with 1-butyl-3-methylimidazolium hexafluorophosphate (C_4_MIM·PF_6_), which exhibited not only good biocompatibility with the cells but also excellent solvent properties for the toxic substrate and product, thus markedly improving the efficiency of the bioreduction and the operational stability of the cells as compared to the IL-free aqueous system. 2-Propanol was shown to be the most suitable co-substrate for coenzyme regeneration, and it was found that the optimum volume ratio of buffer to C_4_MIM·PF_6_, substrate concentration, buffer pH, 2-propanol concentration and reaction temperature were 4/1 (v/v), 24 mM, 5.5, 130 mM and 30°C, respectively. Under these optimized conditions, the maximum yield and the product *e.e*. wer 97.7% and >99%, respectively, which are much higher than the corresponding values previously reported. The efficient whole-cell biocatalytic process was shown to be feasible on a 250-mL scale.

**Conclusion:**

The whole cell-catalyzed asymmetric reduction of TMSB to (*S*)-TMSBOL can be substantially improved by using a C_4_MIM·PF_6_/buffer biphasic system instead of a single-phase aqueous system and the resulting biocatalytic process appears to be effective and competitive on a preparative scale.

## Background

Enantiopure chiral alcohols have proved to be versatile intermediates for the synthesis of many chiral pharmaceuticals, agrochemicals, liquid crystals and flavors [1,2]. As the silicon counterparts of chiral alcohols, enantiopure silicon-containing alcohols are becoming increasingly attractive, in that these silicon-containing compounds play an important role not only in asymmetric synthesis and functional materials, but also in the preparation of silicon-containing drugs [3,4], such as Zifrosilone [5], Cisobitan [6] and TAC-101{4-[3,5-bis(trimethylsilyl)benzamido]benzoic acid} [7]. Such silicon-containing molecules generally have greater pharmaceutical activity, higher selectivity and lower toxicity than their carbon counterparts. The enantiopure organosilicon compound (*S*)-4-(trimethylsilyl)-3-butyn-2-ol {(*S*)-TMSBOL} is a crucial intermediate for the synthesis of 5-lipoxygenase inhibitors [8] for which there is an increasing demand. For economic, environmental and social reasons, production of (*S*)-TMSBOL via biocatalytic asymmetric reduction of the corresponding prochiral 4-(trimethylsilyl)-3-butyn-2-one (TMSB) would be an advantageous alternative to traditional chemical synthesis. Whole microbial cells rather than isolated enzymes would be preferable as the biocatalysts to avoid the need for enzyme purification and coenzyme addition, or the requirement for an additional system for coenzyme regeneration. Also, enzyme inactivation is frequently less of a problem when the enzyme is kept within the natural environments of living cells.

To our knowledge, only one attempt has been made so far to carry out the asymmetric reduction of TMSB to (*S*)-TMSBOL with an isolated enzyme, where yield (78%) and product *e.e*. (57%) were both relatively low [9], possibly due to the low activity and enantioselectivity of the enzyme, as well as possible inhibition of the reaction by substrate and product. Until recently, there has to our knowledge been no report of whole cell-mediated asymmetric reduction of TMSB to enantiopure (*S*)-TMSBOL. Recently we screened a large number of microbial strains for this reaction, including yeasts (*Candida parapsilosis *CCTCC M203011, *Rhodotorula *sp. AS2.2241, *Candida tropicalis, Saccharomyces cerevisiae, Trigonopsis variabilis*), bacteria (*Lactobacillus brevis*, *Bacterium anthracoides*) and a mold (*Geotrichum candidum*) [10]. It was found that *Candida parapsilosis *CCTCC M203011, a highly potent carbonyl reductase-producing organism capable of effectively catalyzing the stereoselective reduction of a variety of prochiral ketones [11,12], was the best strain tested for efficient synthesis of (*S*)-TMSBOL *via *asymmetric reduction of TMSB, in terms of the relatively high yield (81.3%) and the excellent product *e.e*. (>99.9%). However, when the bioreduction was conducted in an aqueous monophasic system, high percentage yield was obtained only when the substrate concentration was = 3 mM [10], due to pronounced substrate and product inhibition of the reaction at higher concentrations. Furthermore, the substrate TMSB is unstable and liable to cleavage into a carbonyl alkyne and trimethylhydroxysilane in aqueous medium. Such cleavage of the substrate can be minimised by decreasing the pH of the aqueous buffer, but this strategy may result in the inactivation of microbial cells. Poor aqueous solubility of hydrophobic ketone substrates may also be a problem in such reactions [13].

In order to overcome these limitations a biphasic system has been developed, where an aqueous buffer contains the microbial cells and a water-immiscible organic phase acts as a reservoir for substrate and product [14-16]. However, use of conventional organic solvents in such processes may be problematic because in many cases they are toxic to the microbial cells. Also, they may be explosive and are usually environmentally harmful. Hydrophobic ionic liquids (ILs) are a promising new class of alternative 'green' solvents that are obvious candidates for a great variety of biocatalytic transformations [17-21]. Many kinds of ILs have proven to be biocompatible with efficient biotransformations catalysed by diverse microbial cells, including *Saccharomyces cerevisiae*, *Escherichia coli*, *Geotrichum candidum*, *Rhodotorula *sp. AS2.2241, *Pichia membranaefaciens *Hansen ZJPH07 and *Lactobcillus kefir *[21-27], since Cull *et al *[28] first reported successful use of the IL 1-butyl-3-methylimidazolium hexafluorophosphate (C_4_MIM·PF_6_) in a biphasic system for the hydrolysis of 1, 3-dicyanobenzene catalyzed by *Rhodococcus *R312 cells. To date, no report has been published on biocatalysis with whole cells of *Candida parapsilosis *CCTCC M203011 in IL-containing systems.

**Figure 1:**
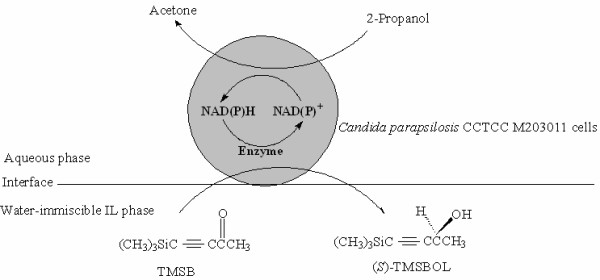
**The bioreduction of TMSB to (*S*)-TMSBOL with immobilized *Candida parapsilosis *CCTCC M203011 cells in water-immiscible IL/buffer biphasic systems**.

In the present study, we have for the first time utilized various water-immiscible ILs in a two-phase system to improve the biocatalytic asymmetric reduction of TMSB to (*S*)-TMSBOL, catalyzed by immobilized *Candida parapsilosis *CCTCC M203011 cells (Fig. 1). In this system, TMSB is reduced to enantiopure (*S*)-TMSBOL while converting NAD(P)H to NAD(P)^+^, and the co-substrate 2-propanol is simultaneously oxidized to acetone, thus driving the reduction reaction by regenerating NAD(P)H from NAD(P)^+^.

## Results and discussion

Up to now, there have been a number of reports on biocatalytic reduction of ketones using microbial cells in various IL-containing reaction systems, where the catalytic performances exhibited by the biocatalysts were closely related to the cation and anion types of ILs, and the effect of various ILs on the biocatalytic reactions has been found to vary widely [25,27,29-31]. Therefore, we initially performed asymmetric reduction of TMSB to (*S*)-TMSBOL, catalyzed by immobilized *Candida parapsilosis *CCTCC M203011 cells, in various IL-based biphasic systems in order to focus on the influence that the cations and the anions of the various ILs had on the bioreduction (Table 1). A variety of ILs were chosen to allow exploration of a range of anionic and cationic moieties. In order to allow efficient use in the biotransformation reaction, ILs were chosen that were liquid at 30°C [23], had density greater than 1.2 g/cm^3 ^(to allow effective separation of the phases after the reaction) [23] and when water-saturated had a viscosity of less than 400 mm^2^/s (in order to minimize mass transfer limitations).

**Table 1:** Effect of various water-immiscible ILs on the bioreduction

Medium	Viscosity of IL (cP, 30°C)	*V*_o_^*a*^ (*μ*mol/min g_cwm_)	Yield^*b*^ (%)	*e.e*.^*c*^ (%)
Aqueous buffer	-	1.18^*d*^	33.6^*d*^	>99^*d*^
C_4_MIM·PF_6_/buffer	173	1.56	85.0	>99
C_5_MIM·PF_6_/buffer	240	1.41	81.2	>99
C_6_MIM·PF_6_/buffer	312	1.30	77.4	>99
C_7_MIM·PF_6_/buffer	355	1.23	72.3	>99
*i*C_4_MIM·PF_6_/buffer	238	1.04	63.1	>99
C_2_MIM·Tf_2_N/buffer	25	1.21	71.6	>99
C_4_MIM·Tf_2_N/buffer	44	1.08	64.7	>99

Reaction conditions: 12 mM TMSB, IL/TEA-HCl buffer (100 mM, pH 5.0) volume ratio of 1/2, 98 mM 2-propanol, 0.15 g/mL cell-loaded alginate beads, 30°C, 180 r/min.

^*a *^Initial reaction rate (*V*_o_) is defined as the initial rate of the product formation in the total reaction system, expressed as the specific activity in *μ*mol product per min per gram of cell wet mass (cwm) unless specified otherwise.

^*b *^Maximum yield.

^*c *^Product *e.e*.

^*d *^Data taken from Zhang *et al *[10].

**Figure 2:**
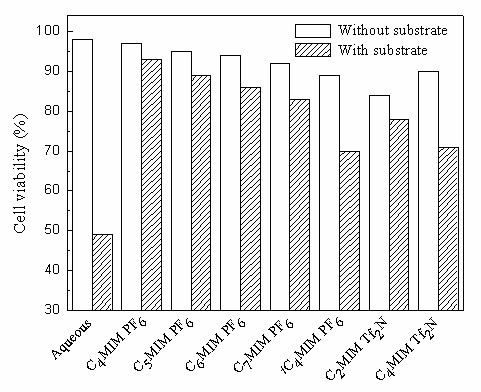
**Cell viability of *Candida parapsilosis *CCTCC M203011**. The cells were exposed for 12 h to various biphasic systems consisting of water-immiscible ILs and TEA-HCl buffer (100 mM, pH 5.0) (IL/buffer volume ratio: 1/2) or aqueous buffer (100 mM, pH 5.0), with and without substrate (12 mM TMSB).

**Figure 3:**
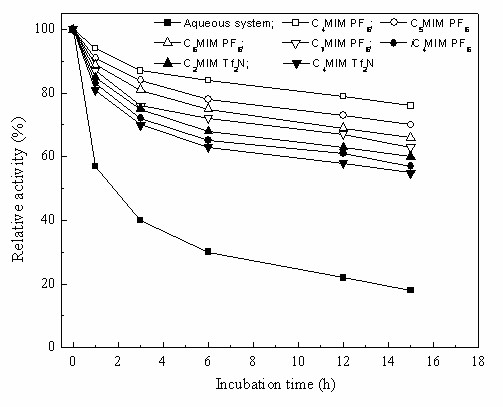
**Deactivation profiles of the cells in the presence of product in various IL-based biphasic systems**. In each case, the 100% relative activity corresponded to the initial activity of the cells.

It was noted that the *Candida parapsilosis *CCTCC M203011 cells were capable of catalyzing the asymmetric reduction of TMSB in the various IL-based biphasic systems with a high product *e.e*. of above 99%. For the biphasic systems involving C_*n*_MIM·PF_6 _(*n *= 4-7), both the initial reaction rate and the maximum yield clearly decreased with the elongation of the alkyl chain (*i.e*. increasing *n *value) of the IL cation, possibly partly because of the increase in viscosity of the IL with increasing *n *value [20,25,32], which may lead to a decrease of substrate and product mass transfer rate between the two phases. Alternatively, both the slightly lower partition coefficients of TMSB and TMSBOL between IL and buffer (Table 2) and the lower biocompatibility of IL with *Candida parapsilosis *CCTCC M203011 cells (Fig. 2) with increasing *n *value could explain this observation. As indicated in Fig. 2, the cell viability was clearly reduced in the presence of substrate as compared to its absence in all the reaction systems, especially in the aqueous monophasic system, implying that the substrate and/or product exerted a substantial toxicity to *Candida parapsilosis *CCTCC M203011 cells. In comparison with IL-based biphasic systems, the cell viability in the presence of substrate was much lower in the aqueous monophasic system, which is in good accordance with the higher initial specific reaction rate and yield in the presence of most of the tested ILs (C_*n*_MIM·PF_6_, *n *= 4-7; C_2_MIM·Tf_2_N), as compared to the aqueous system (Table 1). It was noted that the biphasic system containing *i*C_4_MIM·PF_6 _or C_4_MIM·Tf_2_N gave a slightly lower initial reaction rate than the aqueous system, but afforded a clearly higher yield. Higher partition coefficients of TMSB and TMSBOL between IL and buffer could effectively reduce the toxic effect of the substrate and/or the product on the cells as well as the pronounced inhibitions of the reaction by the substrate and the product observed in aqueous monophasic system [10]. To better understand the toxic or inhibitory effects of the product in the various IL-based biphasic systems, the deactivation profiles of the cells in different media in the presence of 12 mM TMSBOL product were investigated (Fig. 3). After incubation in the aqueous system with substrate for 1 h and 15 h, the cells retained only 58% and 17%, respectively, of their original activity, clearly showing the severe toxic or inhibitory effect of the product. However, the cells in IL-based biphasic systems retained much higher relative activity (as compared to the cells in the aqueous monophasic system) after incubation for a same period. Among the seven water-immiscible ILs tested in the biphasic reaction system, in all cases decreasing rate of inactivation of the biocatalyst correlated with increasing product IL/aqueous buffer partition coefficient. This observation suggests that a major factor in determining the stability of the biocatalytic activity of the cells is extraction of the toxic reaction product into the IL phase. The biphasic system containing C_4_MIM·PF_6 _was most effective in preserving the activity of the biocatalyst and contained the IL with the highest product IL/aqueous buffer partition coefficient. In addition to the likely toxic effect of the product, the data in Fig. 2 suggest that the substrate also is toxic to the cells in the presence of ILs.

**Table 2:** Partition coefficients of TMSB and TMSBOL between the two phases

Biphasic system	Partition coefficients	
	
	TMSB	TMSBOL
C_4_MIM·PF_6_/buffer	56.3	26.2
C_5_MIM·PF_6_/buffer	55.7	25.1
C_6_MIM·PF_6_/buffer	54.2	24.4
C_7_MIM·PF_6_/buffer	53.7	23.8
*i*C_4_MIM·PF_6_/buffer	50.9	21.6
C_2_MIM·Tf_2_N/buffer	53.4	23.1
C_4_MIM·Tf_2_N/buffer	51.1	20.3

In the case of the Tf_2_N- -based ILs (C_2_MIM·Tf_2_N and C_4_MIM·Tf_2_N), the initial reaction rate and the maximum yield decrease with increasing alkyl chain, showing a similar trend to that observed with C_*n*_MIM·PF_6 _(*n *= 4-7). The partition coefficients of TMSB and TMSBOL in the C_2_MIM·Tf_2_N/buffer biphasic system are higher than the corresponding values in the C_4_MIM·Tf_2_N/buffer biphasic system (Table 2), which is consistent with the observation that in the presence of substrate the cell viability is somewhat higher in the C_2_MIM·Tf_2_N-contianing system than in that containing C_4_MIM·Tf_2_N (Fig. 2). Also, it should be noted that in the absence of substrate, C_4_MIM·Tf_2_N is less toxic to the microbial cells than C_2_MIM·Tf_2_N (Fig. 2). Conversely, the higher partition coefficients of TMSB and TMSBOL with C_2_MIM·Tf_2_N (relative to C_4_MIM·Tf_2_N) will tend to favor an efficient reduction reaction by diminishing the concentrations of the substrate and product in the aqueous phase and thus reducing their inhibitory effects on the cells. This is the reason that the initial reaction rate and the yield achieved with the C_2_MIM·Tf_2_N-based biphasic system were better than those with the system containing C_4_MIM·Tf_2_N (Table 1), even though the former IL is marginally more toxic to the cells. Additionally, the biocatalytic reduction proceeded more slowly in the presence of C_4_MIM·Tf_2_N than in the presence of C_4_MIM·PF_6_, showing that the nature of the anion in the ILs has a significant effect on the bioreduction. It was worth noting that the initial reaction rate and the maximum yield clearly went down when the *n*-butyl group attached to the imidazolium cation of C_4_MIM·PF_6 _was replaced by *iso*-butyl (*i*C_4_MIM·PF_6_). This suggests that the variation of IL structure also exerts a substantial impact on the bioreduction. Interestingly, this minor change of IL structure from C_4_MIM·PF_6 _to *i*C_4_MIM·PF_6 _also led to a decline in the cell viability (Fig. 2) and a fall in the partition coefficients of substrate and product between the IL phase and the aqueous phase (Table 2), which coincides with the clearly poorer catalyst performance achieved in the *i*C_4_MIM·PF_6_-buffer biphasic system. Moreover, the specific reaction rate (1.04-1.56 *μ*mol/min g_cwm_) in the IL-based biphasic systems examined was comparable to or higher than that in the aqueous monophasic system (1.18 *μ*mol/min g_cwm_), and the maximum yield (63.1-85.0%) was much higher than that in the aqueous monophasic system (33.6%) under the same reaction conditions. This superior performance of the biocatalyst in the IL-containing system is probably due to the markedly reduced toxic effect of the substrate to the cells, limited substrate cleavage, and product and/or substrate inhibition, since the ILs effectively extract substrate and product from the aqueous phase. As depicted in Fig. 2, in the absence of substrate, the cell viability was lower in all tested IL-based biphasic systems, compared to the aqueous monophasic system. This indicates that the ILs were toxic to the cells to some extent. Among all the water-immiscible ILs tested, the IL C_4_MIM·PF_6 _afforded the highest cell viability (94%) (Fig. 2) and the highest partition coefficients for TMSB and TMSBOL (Table 1), and therefore the highest yield and specific reaction rate were observed in the C_4_MIM·PF_6_-based biphasic system. Clearly, C_4_MIM·PF_6 _was the best second phase for the bioreduction.

**Figure 4:**
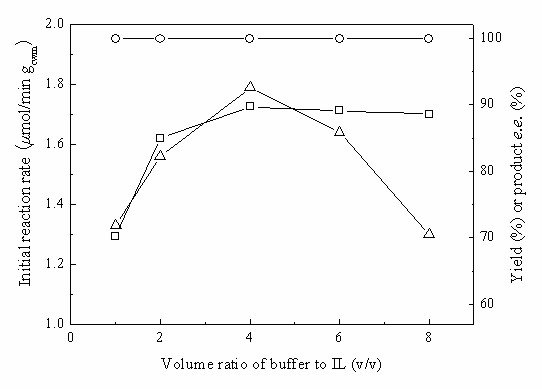
**Effect of volume ratio of buffer to IL on the biocatalytic reduction of TMSB with immobilized *Candida parapsilosis *CCTCC M203011 cells**. Reaction conditions: 12 mM TMSB, 0.5 mL of C_4_MIM·PF_6_, various volumes of TEA-HCl buffer (100 mM, pH 5.0), 98 mM 2-propanol, 0.15 g/mL cell-loaded alginate beads, 30°C, 180 r/min. *Symbols*: (○) product *e.e*.; (□) maximum yield; (Δ) initial reaction rate.

For a better understanding of the bioreduction performed in the biphasic system containing C_4_MIM·PF_6_, the effects of several key influential variables on the reaction were studied. In our previous reports, it has been demonstrated that the effect of volume ratio of the two phases on biocatalytic reactions varies widely and unpredictably [22,25]. As shown in Fig. 4, the volume ratio of the aqueous phase to the IL phase (V_aq_/V_IL_, mL/mL) substantially affected the initial reaction rate and the maximum yield, but had no appreciable effect on the product *e.e*. The obvious enhancement in the initial reaction rate and the maximum yield with the increase of V_aq_/V_IL _up to 4/1 suggests inactivation of the catalytic activity of the cells that is less pronounced as the proportion of IL in the mixture decreases over this range. Enzymes and active cells are often inactivated by direct contact with the interface between the aqueous and non-aqueous phases [25,33], and if this were the case here, the increase of activity and yield between V_aq_/V_IL _of 1/1 and 4/1 could be due to the decrease in the interface area of the two-liquid phase reaction system and consequent decreased frequency with which the cells would contact the interface. However, such a phenomenon may be unlikely since the ILs showed low toxicity to the cells (Fig. 2). A more likely possibility is that the effects of substrate toxicity or inhibition decrease as the rate of substrate delivery into the aqueous phase decreases with decreasing interface area between the phases. Further rise in the V_aq_/V_IL _ratio (above the optimal 4/1 value) led to a clear decline in the initial reaction rate, presumably due to much lower availability of the substrate in the aqueous phase. Another possibility is that cell inactivation at the interface can occur because of toxicity of highly concentrated substrate rather than the IL itself.

**Figure 5:**
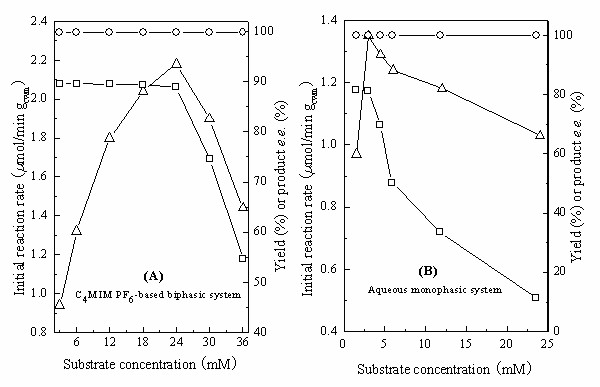
**Effect of substrate concentration on the biocatalytic reduction of TMSB with immobilized *Candida parapsilosis *CCTCC M203011 cells in the C_4_MIM·PF_6_-based system (A) and in the aqueous system (B)**. Reaction conditions: (A): various concentrations of TMSB, C_4_MIM·PF_6_/TEA-HCl buffer (100 mM, pH 5.0) volume ratio of 1/4 (0.5 mL/2.0 mL), 98 mM 2-propanol, 0.15 g/mL cell-loaded alginate beads, 30°C, 180 r/min; (B): various concentrations of TMSB, TEA-HCl buffer (4 mL, 100 mM, pH 5.0), 98 mM 2-propanol, 0.15 g/mL cell-loaded alginate beads, 30°C, 180 r/min. *Symbols*: (○) product *e.e*.; (□) maximum yield; (Δ) initial reaction rate.

As depicted in Fig. 5A, the initial reaction rate of the bioreduction conducted in the C_4_MIM·PF_6_-based biphasic system markedly increased with increasing substrate concentration up to 24 mM (based on the volume of the IL phase), while the maximum yield showed no significant variation. Further rise in substrate concentration above 24 mM, however, led to a clear drop in the initial rate and the maximum yield. The results clearly show that the inhibitory or toxic effect of the substrate begins to dominate if the substrate concentration added to the IL phase is more than 24 mM, although below this threshold it is clear that the substrate concentration in the aqueous phase is limiting. Throughout the range of substrate concentrations tested the product *e.e*. remained above 99%. Fig. 5B shows the significant influence of substrate concentration on the bioreduction carried out in the aqueous monophasic system. When the substrate concentration was more than 3 mM, the initial reaction rate and the maximum yield decreased substantially with increasing substrate concentration, showing that substrate inhibition or toxicity occurs even if the applied substrate concentration in the aqueous monophasic system is very low (3 mM). Product inhibition also occurred in the C_4_MIM·PF_6_-based system, because the reaction rate and the maximum yield decreased with increasing concentration of added product when a range of TMSBOL concentrations were added at the beginning of the reaction (Table 3). However, product inhibition was less pronounced in the C_4_MIM·PF_6_-based system as compared to the aqueous system [10]. The optimal substrate concentration (24 mM) in the C_4_MIM·PF_6_-based system is much higher than that (3 mM) in the aqueous monophasic system.

**Table 3:** Effect of TMSBOL addition on the bioreduction conducted in C_4 _MIM·PF_6_/buffer biphasic system

Addition of TMSBOL (mM)	*V*_o_ (*μ*mol/min g_cwm_)	Yield^*a*^ (%)	*e.e*.^*b*^ (%)
0	2.18	89.6	>99
3	1.88	84.5	>99
6	1.67	78.1	>99
12	1.34	68.8	>99
24	0.75	33.7	>99

Reaction conditions: 24 mM TMSB, C_4_MIM·PF_6_/TEA-HCl buffer (100 mM, pH 5.0) volume ratio of 1/4, 98 mM 2-propanol, 0.15 g/mL cell-loaded alginate beads, 30°C, 180 r/min.

^*a *^Maximum yield.

^*b *^Product *e.e*.

**Figure 6:**
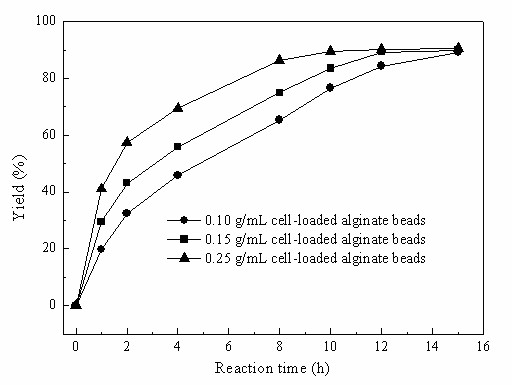
**Effect of biocatalyst concentration on the biocatalytic reduction of TMSB with immobilized *Candida parapsilosis *CCTCC M203011 cells**. Reaction conditions: 24 mM TMSB, C_4_MIM·PF_6_/TEA-HCl buffer (100 mM, pH 5.0) volume ratio of 1/4, 98 mM 2-propanol, various concentrations of cell-loaded alginate beads (based on the volume of the aqueous phase), 30°C, 180 r/min.

As can be seen in Fig. 6, the reaction rate of the substrate TMSB (24 mM) reduction in the C_4_MIM·PF_6_-based biphasic system clearly increased with increasing concentration of cell-loaded alginate beads (0.10 - 0.25 g/mL, based on the volume of the aqueous phase). Therefore, it could be concluded that there is no clear mass transfer limitation over the two-phase boundary under the above-mentioned reaction conditions.

**Figure 7:**
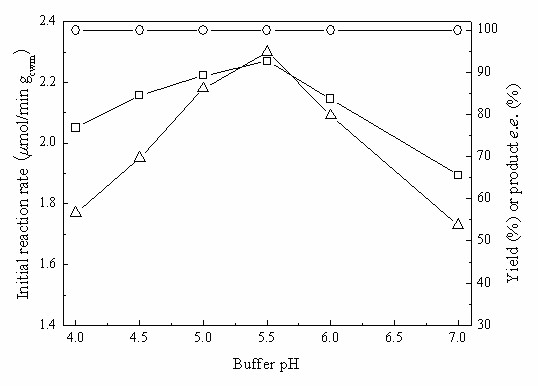
**Effect of buffer pH on the biocatalytic reduction of TMSB with immobilized *Candida parapsilosis *CCTCC M203011 cells**. Reaction conditions: 24 mM TMSB, C_4_MIM·PF_6_/TEA-HCl buffer (100 mM, various pHs) volume ratio of 1/4, 98 mM 2-propanol, 0.15 g/mL cell-loaded alginate beads, 30°C, 180 r/min. *Symbols*: (○) product *e.e*.; (□) maximum yield; (Δ) initial reaction rate.

Fig. 7 illustrates the significant effect of buffer pH in the C_4_MIM·PF_6_/buffer biphasic system on the bioreduction and shows that the optimal buffer pH for the reaction was pH 5.5. Throughout the range of buffer pH tested, the product *e.e*. remained above 99%.

**Figure 8:**
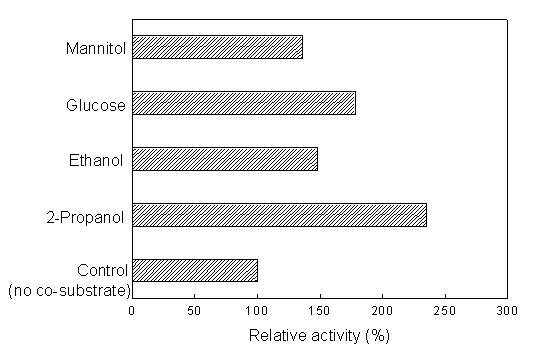
**Effect of various co-substrates on the biocatalytic reduction of TMSB with immobilized *Candida parapsilosis *CCTCC M203011 cells**. Reaction conditions: 24 mM TMSB, C_4_MIM·PF_6_/TEA-HCl buffer (100 mM, pH 5.5) volume ratio of 1/4, different co-substrates (98 mM), 0.15 g/mL cell-loaded alginate beads, 30°C, 180 r/min. The relative activity of the cells without any co-substrates was defined as 100%.

**Figure 9:**
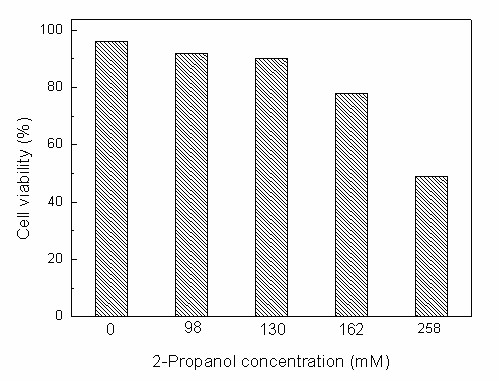
**Cell viability of *Candida parapsilosis *CCTCC M203011 in the presence of 2-propanol**. The cells were exposed for 12 h to the medium containing various concentrations of 2-propanol.

Coenzyme recycling is one of the most important issues encountered in biocatalytic reduction reactions. Bioreduction reactions can proceed effectively with whole cells without adding expensive coenzymes only if a co-substrate is present for recycling of the coenzyme [34]. Therefore several commonly-employed co-substrates (2-propanol, ethanol, glucose, mannitol) were tested. As shown in Fig. 8, although TMSB could be reduced without adding co-substrates into the reaction system (control), the maximum yield was only 25.6%. Adding any single co-substrate markedly improved the bioreduction, with 2-propanol giving the highest activity. It has been reported that, in addition to its role as a co-substrate, 2-propanol might act as a permeabilizer moderately to increase the cell membrane permeability [35], and thus allow substrate and product to pass more quickly in and out of cells, thus accelerating the bioreduction reaction. As is evident from the data shown in Table 4, the initial reaction rate and the maximum yield increased with increasing 2-propanol concentration up to 130 mM, beyond which further rise in 2-propanol concentration resulted in a clear decrease in the initial reaction rate and the maximum yield, possibly because of the negative effects of the excessive 2-propanol on the cells. To test this possibility, the viability of *Candida parapsilosis *CCTCC M203011 cells was assayed after 12 h exposure to the biphasic systems containing various concentrations of 2-propanol. As expected, cell viability showed no appreciable decline with increasing 2-propanol concentration up to 130 mM. However, further rise in 2-propanol concentration led to a rapid loss of cell viability (Fig. 9). The 2-propanol concentration showed little influence on the product *e.e*., which remained above 99% within the range tested. Thus, the optimal concentration of 2-propanol for the reaction was 130 mM.

**Table 4:** Effect of co-substrate concentration on the bioreduction

2-Propanol concentration (mM)	*V*_o_ (*μ*mol/min g_cwm_)	Yield^*a*^ (%)	*e.e*.^*b*^ (%)
0	0.95	25.6	>99
34	1.57	72.9	>99
66	1.99	85.1	>99
98	2.30	92.8	>99
130	2.46	97.7	>99
162	2.22	90.5	>99
194	1.88	80.3	>99
258	1.07	50.4	>99

Reaction conditions: 24 mM TMSB, C_4_MIM·PF_6_/TEA-HCl buffer (100 mM, pH 5.5) volume ratio of 1/4, various concentrations of 2-propanol, 0.15 g/mL cell-loaded alginate beads, 30°C, 180 r/min.

^*a *^Maximum yield.

^*b *^Product *e.e*.

**Figure 10:**
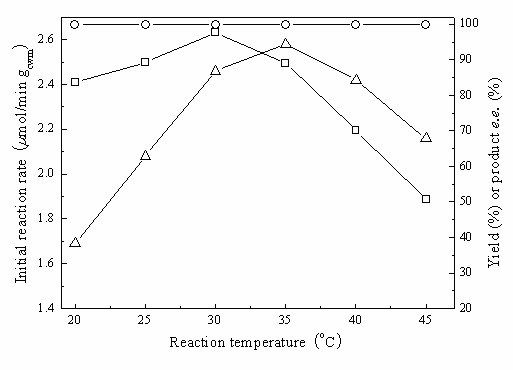
**Effect of reaction temperature on the biocatalytic reduction of TMSB with immobilized *Candida parapsilosis *CCTCC M203011 cells**. Reaction conditions: 24 mM TMSB, C_4_MIM·PF_6_/TEA-HCl buffer (100 mM, pH 5.5) volume ratio of 1/4, 130 mM 2-propanol, 0.15 g/mL cell-loaded alginate beads, various temperatures, 180 r/min. *Symbols*: (○) product *e.e*.; (□) maximum yield; (Δ) initial reaction rate.

As can be seen in Fig. 10, the specific reaction rate increased with increasing reaction temperature up to 35°C, while the maximum yield clearly decreased above 30°C, owing to the inactivation of the cells that had been incubated for a prolonged period at a higher temperature. The product *e.e*. remained above 99% within the tested range. In terms of maximising reaction rate, the reaction yield and the product *e.e*., 30°C was concluded to be as the most suitable temperature for the reaction.

**Figure 11:**
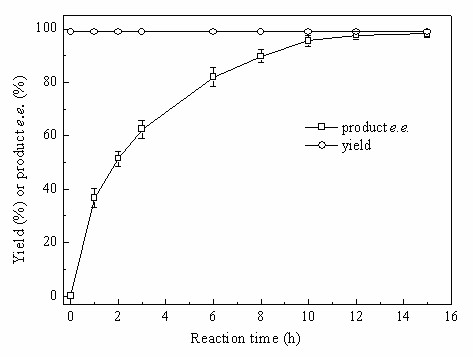
**Time-course profile of the biocatalytic reduction of TMSB with immobilized *Candida parapsilosis *CCTCC M203011 cells in C_4_MIM·PF_6_/buffer biphasic system**. Reaction conditions: 24 mM TMSB, C_4_MIM·PF_6_/TEA-HCl buffer (100 mM, pH 5.5) volume ratio of 1/4, 130 mM 2-propanol, 0.15 g/mL cell-loaded alginate beads, 30°C, 180 r/min.

Fig. 11 depicts the time-course of the bioreduction of TMSB with immobilized *Candida parapsilosis *CCTCC M203011 cells in the C_4_MIM·PF_6_-based biphasic system under the above-described optimum conditions. The reaction rate decreased relatively slowly with reaction time in the C_4_MIM·PF_6_-based system as compared to the aqueous system [10], almost certainly due to the *in situ *extraction of substrate and product into the IL phase. Therefore, the reaction efficiency was substantially enhanced with C_4_MIM·PF_6 _as compared to the aqueous system and the yield was increased from 81.3% with the aqueous system [10] to 97.7% in the IL-containing system. The product *e.e*. remained above 99% in both reaction systems.

To show the applicability of the biocatalytic reduction of TMSB to (*S*)-TMSBOL using immobilized *Candida parapsilosis *CCTCC M203011 cells in the C_4_MIM·PF_6_-based biphasic system, we also carried out the bioreduction on a 250-mL preparative scale under the optimal reaction conditions detailed above, i.e. 24 mM TMSB, C_4_MIM·PF_6_/TEA-HCl buffer (100 mM, pH 5.5) volume ratio of 1/4 (50 mL/200 mL), 130 mM 2-propanol, 0.15 g/mL cell-loaded alginate beads, 30°C, 180 r/min. The reaction process was monitored by GC analysis and the product was extracted from the reaction mixture with *n*-hexane upon the exhaustion of the substrate. The bioreduction behavior was similar to that shown in Fig. 11. Although slightly lower than that obtained on the 2.5-mL scale, the isolated yield (95.9%) after reaction for 15 h on the 250-mL scale was much higher than that achieved in aqueous system [9,10], and the product *e.e*. was excellent (>99%). Furthermore, no emulsification of the IL-based biphasic system was observed, so the phases could be separated readily by centrifugation. No by-products accumulated in the IL phase, and the IL could be easily recycled, reducing the overall cost of the biocatalytic process. Hence the whole-cell biocatalytic reduction of TMSB to (*S*)-TMSBOL on a preparative scale in the C_4_MIM·PF_6_-based biphasic system is a promising and competitive reaction.

**Figure 12:**
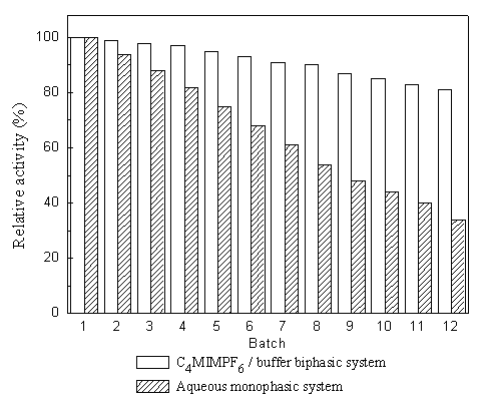
**Operational stability of immobilized *Candida parapsilosis *CCTCC M203011 cells**. Reaction conditions with C_4_MIM·PF_6_/buffer biphasic system: 24 mM TMSB, C_4_MIM·PF_6_/TEA-HCl buffer (100 mM, pH 5.5) volume ratio of 1/4, 130 mM 2-propanol, 0.15 g/mL immobilized cells, 30°C, 180 r/min, 12 h per batch. Reaction conditions with aqueous system: 3 mM TMSB, 2.5 mL of TEA-HCl buffer (100 mM, pH 5.0), 65.3 mM 2-propanol, 0.15 g/mL cell-loaded alginate beads, 30°C, 180 r/min, 1 h per batch. The relative activity of the immobilized cells in the first batch was defined as 100%.

The operational stability of the biocatalyst was also investigated in the C_4_MIM·PF_6_-based biphasic system, in comparison with the aqueous system. As depicted in Fig. 12, after being used repeatedly for 1 batch (12 h per batch) in the presence of C_4_MIM·PF_6_, the immobilized cells retained around 99% of their original activity, which was much higher than that in the aqueous system (34%) during the same operation period (12 batches, 1 h per batch). Furthermore, the immobilized cells still retained more than 83% of their initial activity even after being used repeatedly for 12 batches in the C_4_MIM·PF_6_-based biphasic system. Hence it is clear that C_4_MIM·PF_6 _considerably enhances the operational stability of the cells. This enhancement of the operational stability of the cells may be due to the very good biocompatibility of C_4_MIM·PF_6 _and its excellent ability to extract toxic substrate and product from the aqueous phase. The cells maybe also become coated with the IL and thus protected from the inactivation. In addition, the interactions between the IL and the carrier (calcium alginate) used for immobilization of the cells could contribute to the good stability of the cells in the C_4_MIM·PF_6_-based biphasic system.

## Conclusion

The synthesis of (*S*)-TMSBOL can be successfully conducted with high yield and excellent product *e.e*. by means of the biocatalytic asymmetric reduction of TMSB using immobilized *Candida parapsilosis *CCTCC M203011 cells in water-immiscible IL-based biphasic systems. Various ILs exerted significant but different influences on the bioreduction. Of all the examined ILs, C_4_MIM·PF_6 _was most effective for the bioreduction. The optimal substrate concentration (24 mM *vs *3 mM) and the yield (97.7% *vs *81.3) were substantially enhanced in the C_4_MIM·PF_6_-based system compared to the aqueous system, and the cells also retained a much higher relative activity in the C_4_MIM·PF_6_-based system than that in the aqueous system after being employed repeatedly for 12 batches (83% *vs *34%), showing the excellent operational stability in the presence of C_4_MIM·PF_6_. The good performance of the biocatalyst in the presence of the IL may be due to the IL's excellent solvent properties for substrate and product and its good biocompatibility with the cells. Furthermore, the results described here clearly show that the whole-cell biocatalytic process in the presence of C_4_MIM·PF_6 _is feasible up to a 250-mL scale. If further scale-up is possible, the reaction will be attractive for large-scale industrial application.

## Methods

### Biological and chemical materials

*Candida parapsilosis *CCTCC M203011 was kindly donated by Professor Yan Xu (Key Laboratory of Industrial Biotechnology of Ministry of Education and School of Biotechnology, Southern Yangtze University, China).

4-(Trimethylsilyl)-3-butyn-2-one (TMSB, 97% purity), 4-(trimethylsilyl)-3-butyn-2-ol (TMSBOL, 97% purity) and *n*-decane (>99% purity) were purchased from Sigma-Aldrich (USA). The seven water-immiscible ILs used in this work, 1-butyl-3-methylimidazolium hexafluorophosphate (C_4_MIM·PF_6_), 1-pentyl-3-methylimidazolium hexafluorophosphate (C_5_MIM·PF_6_), 1-hexyl-3-methylimidazolium hexafluorophosphate (C_6_MIM·PF_6_), 1-heptyl-3-methylimidazolium hexafluorophosphate (C_7_MIM·PF_6_), 1-*iso*butyl-3-methylimidazolium hexafluorophosphate (*i*C_4_MIM·PF_6_), 1-ethyl-3-methylimidazolium bis(trifluoromethanesulfonyl)imide (C_2_MIM·Tf_2_N) and 1-butyl-3-methylimidazolium bis(trifluoromethanesulfonyl)imide (C_4_MIM·Tf_2_N) were from Lanzhou Institute of Chemical Physics (China) and were all of over 97% purity. All other chemicals were obtained from commercial sources and were of analytical grade.

### Cultivation and immobilization of Candida parapsilosis CCTCC M203011 cells

*Candida parapsilosis *CCTCC M203011 cells were cultivated and immobilized as described in [10].

### General procedure for the bioreduction of TMSB to (S)-TMSBOL

In a typical experiment, the biphasic system (2.5 mL) consisted of a water-immiscible IL and TEA-HCl buffer (100 mM, various pHs: 4.0-7.0), contained in a 20-mL Erlenmeyer flask capped with a septum. Alginate beads were prepared that were loaded with 31% (w/w) *Candida parapsilosis *CCTCC M203011 cells {based on cell wet mass (cwm)}and 0.15 g of these cell-loaded alginate beads were added per mL of the aqueous phase, together with a predetermined quantity of co-substrate (0-258 mM based on the total volume of a biphasic system). The reaction mixture was pre-incubated in a water-bath shaker at 180 r/min and various temperatures (20-45°C) for 15 min. Then, the reactions were initiated by adding TMSB at various concentrations (3-36 mM, based on the volume of the IL phase). Aliquots (10 *μ*L) were withdrawn at specified time intervals from the IL phase and the aqueous phase, respectively, and the product as well as the residual substrate was extracted with *n*-hexane (50 *μ*L) containing 5.1 mM *n*-decane (as an internal standard), prior to GC analysis. Details of the IL used, volume ratio of buffer to IL, substrate concentration, buffer pH, co-substrate and its concentration and reaction temperature are specified for each case. Throughout this paper, unless specified otherwise, applied substrate concentration refers to that in the IL phase of a biphasic system and the co-substrate concentration is given for the total volume of a biphasic system.

### Cell viability assay

The viability of *Candida parapsilosis *CCTCC M203011 cells was assayed after incubating the alginate-immobilized cells for 12 h in various biphasic systems consisting of water-immiscible ILs and TEA-HCl buffer (100 mM, pH 5.0) (IL/buffer volume ratio: 1/2), or TEA-HCl buffer (100 mM, pH 5.0) system, without substrate and with substrate (12 mM TMSB, based on the volume of the IL phase), respectively. The beads containing the immobilized cells were withdrawn from the reaction systems and then added to 0.1 M trisodium citrate to dissolve the alginate. After this, the microbial cell suspension was diluted and stained with 0.1% Methylene Blue for 5 min [25,29]. Microscopic pictures were taken and analyzed for blue dead cells and colorless viable ones.

### Determination of partition coefficients

Partition coefficients (*K*_*IL*/*aq*_) were determined by dissolving 12, 24 or 36 mM TMSB or TMSBOL, as appropriate, in each IL/buffer biphasic system (IL/buffer volume ratio: 1/2) and shaking (180 r/min) for 40 h at 30°C. The concentrations of TMSB or TMSBOL in the IL phase and the aqueous phase were then analyzed by GC. The concentration of TMSB or TMSBOL in each phase varied linearly with the total amount of each chemical added to the two-phase system. Then the slopes were calculated and used for the quantification of the partition coefficients of TMSB and TMSBOL between the IL phase and the aqueous phase.

### Operational stability of immobilized Candida parapsilosis CCTCC M203011 cells

In order to assess the operational stability of the cells, the re-use of the immobilized *Candida parapsilosis *CCTCC M203011 cells was investigated in the C_4_MIM·PF_6_/buffer biphasic system and also in the aqueous monophasic system. Initially, aliquots of the cells were added into separate screw-capped vials each containing 2.5 mL of the appropriate medium {C_4_MIM·PF_6_/TEA-HCl buffer (100 mM, pH 5.5) biphasic system (volume ratio: 1/4), or aqueous TEA-HCl buffer system (100 mM, pH 5.0)}, together with the optimal amount of TMSB and 2-propanol for the reduction conducted in the various media. Then, the bioreductions were carried out at 30°C and 180 r/min and were repeated over 12 batches without changing the immobilized cells. Between batches, the immobilized cells were filtered off from the reaction mixture, washed twice with fresh water, and added to a fresh batch of reaction medium. The reduction activity of the cells was assayed in each batch. The relative activity of the cells employed for the first batch was defined as 100%.

### GC analysis

Reaction mixtures were analyzed according to the GC analysis method previously reported [10]. The retention-times for TMSB, *n*-decane and TMSBOL were 5.1, 5.7, and 10.5 min, respectively. Also, the product configuration was confirmed to be (*S*)-TMSBOL [10]. The average error for this determination was less than 1.0%. All reported data were averages of experiments performed at least in duplicate.

## Authors' contributions

WYL and MHZ designed the study, analyzed the experiment data and drafted the manuscript. LC and BBZ carried out the experiments and GC analysis, and participated in the design of the study. TJS assisted with data interpretation of the study, and participated in its design and coordination. All authors read and approved the final manuscript.
